# Regulation of cell fate determination in plants

**DOI:** 10.3389/fpls.2014.00368

**Published:** 2014-07-28

**Authors:** Shucai Wang, John Schiefelbein

**Affiliations:** ^1^Key Laboratory of Molecular Epigenetics of Ministry of Education, Key Laboratory of Vegetation Ecology of Ministry of Education, Northeast Normal UniversityChangchun, China; ^2^Department of Molecular, Cellular, and Developmental Biology, University of MichiganAnn Arbor, MI, USA

**Keywords:** cell fate determination, trichome, root hair, stomata, xylem, cotton fiber, protein lipid modification, Arabidopsis

Building a multicellular organism, like a plant, from a single cell requires the coordinated formation of different cell types in a spatiotemporal arrangement. How different cell types arise in appropriate places and at appropriate times is one of the most intensively investigated questions in modern plant biology. Using models such as trichome formation, root hair formation, and stomatal development in Arabidopsis, scientists have begun to discover some of the answers, including the importance of transcriptional regulatory networks, intrinsic signals such as plant hormones, and extrinsic signals such as environmental stimuli. This research topic aimed to summarize the research progress in cell fate determination in plants.

Thanks to the efforts of many people, including the authors who responded enthusiastically to the call to contribute to this research topic, as well as the peer reviewers who provided essential critical comments to ensure the highest quality and up-to-date information in the articles, a total of 12 articles were published in this research topic, including Opinion, Mini Review, Review and Original Research Articles. These articles focused on cell fate determination of different cell types as well as different aspects of a certain cell type in plants.

The specification of distinct cell types in plants is accomplished largely via the establishment of different gene expression, primarily, transcription factor gene expression. For example, recent studies have revealed that certain members of the HD-ZIP class IV homeodomain transcription factors are likely to be master regulators of specification of the shoot epidermal cell layer in plants. Takada and Iida (2014) summarized in their Mini Review the roles of these regulatory genes that are involved in epidermal cell fate specification and discussed the possible mechanisms that limit the expression and/or activity of the HD-ZIP class IV homeodomain genes to the outermost cell layer during the development of plant shoots.

Trichomes and root hairs are specialized epidermal cells whose formation is regulated through a combination of endogenous developmental programs and external signals. In Arabidopsis, trichome and root hair specification is controlled by the interplay of single-repeat R3 MYBs and several other transcription factors including the WD40-repeat protein TTG1, the R2R3 MYB transcription factor GL1 or WER, the bHLH transcription factor GL3 or EGL3, and the homeodomain protein GL2. The TTG1, GL1, or WER, and GL3 or EGL3 proteins are proposed to form a TTG1–GL3/EGL3–GL1/WER activator complex to regulate the expression of GL2, which is required for trichome formation in shoots and non-hair cell specification in roots. On the other hand, the R3 MYB proteins negatively regulate trichome formation and non-hair cell specification by competing with GL1 or WER for binding GL3 or EGL3, thus blocking the formation of TTG1–GL3/EGL3–GL1/WER activator. The expression of component genes of the transcriptional activator complex is regulated by other transcription factors, plant hormones, microRNAs, as well as the 26S proteasome.

Several of the Review and Mini Review articles were devoted to the trichome or root hair cell determination systems. Pattanaik et al. (2014) summarized the gene regulator networks controlling trichome development in Arabidopsis, including the activator complex, their regulation by plant hormones, microRNA and the proteasome system. Hauser (2014) focused on current progress on the molecular basis of the natural variation in various Arabidopsis ecotypes as well as in different plant species with emphasis on plant hormones and environmental stimuli on trichome patterning. Schiefelbein et al. (2014) described the regulatory network and the importance of the multiple feedback loops in root hair cell specification in Arabidopsis, with focus on the mechanisms that lead to the accumulation of the WER-bHLH-TTG1 activate complex in non-hair cells. Tominaga-Wada and Wada (2014) described their findings that tomato and Arabidopsis likely use similar transcription factors for root hair cell differentiation, and that a CPC-like R3 MYB may be a key common regulator of plant root-hair development. In addition, Wang and Chen (2014) focused on the roles of single-repeat R3 MYB transcription factors in the regulation of cell fate determination in Arabidopsis.

In their Original Research Article, Zhou et al. (2014) described the identification and characterization of poplar single-repeat R3 MYB transcription factors in trichome formation. They found a total of eight genes in poplar encoding R3 MYB transcription factors, and all these R3 MYB genes negatively regulate trichome formation when expressed in Arabidopsis.

Stomata are also specialized epidermal cells, and like the formation of trichomes and root hairs, stomatal development is controlled by both an intrinsic genetic regulatory network and environmental stimuli. In Arabidopsis, stomatal precursor cells undergo at least one asymmetric division and a symmetric division. Le et al. (2014) described in their Mini Review the roles of the ER/TMM signal transduction pathway in the regulation of stomata formation and cell cycle, and the role of different plant hormones in stomata development. In her Opinion article, Serna (2003) focused on the regulation of the choice between meristemoid cell self-renewal, in which one of daughters of a dividing meristemoid retains the properties of the parent cell, and its transition through guard mother cell (GMC) fate to produce stomata.

Xylem, phloem, and procambial/cambial cells are differentiated in a spatiotemporally organized manner during vascular development. Various key regulators for xylem cell patterning and differentiation have been discovered, such as the plant hormones auxin and cytokinin, the peptide hormone CLE, microRNAs, and the transcription factor HD-ZIPIIIs, VNDs, SHR, and AHLs. Recent studies revealed that xylem cell fate determination is controlled by functional interactions among these key regulators. Kondo et al. (2014) reviewed the networks of various regulators underlying xylem cell fate determination in root vascular development.

MYBMIXTA transcription factors are involved in the regulation of epidermal cell differentiation in different plant species, including cell shape specification in petals, trichome initiation and branching in shoots and fiber initiation in seeds. MYBMIXTA-like (MML) transcription factors from the subgroup 9 of R2R3-MYBs were first characterized in *Antirrhinum majus*. Bedon et al. (2014) provided evidence in their Opinion article that members of the MML transcription factors regulate the initiation of fiber development in cotton seeds.

Post-translational modifications of proteins are often important for regulation of their functions. One of the key modifications is the attachment of a lipid group to certain amino acids in the proteins, which typically facilitates subcellular targeting and/or protein–protein interactions. Running (2014) summarized in his Review the progress of three known types of intracellular protein lipid modifications, and their roles in many plant-specific aspects of developmental processes, including cell differentiation.

In a summary, we hope the articles in this research topic will provide readers a snapshot of current molecular and genetic investigations of cell fate determination in plants.

## Conflict of interest statement

The authors declare that the research was conducted in the absence of any commercial or financial relationships that could be construed as a potential conflict of interest.
